# Adaptive Multiobjective Evolutionary Generative Adversarial Network for Metaverse Network Intrusion Detection

**DOI:** 10.34133/research.0665

**Published:** 2025-04-15

**Authors:** Dikai Xu, Bin Cao

**Affiliations:** ^1^ State Key Laboratory of Reliability and Intelligence of Electrical Equipment, Hebei University of Technology, Tianjin 300401, China.; ^2^ School of Artificial Intelligence, Hebei University of Technology, Tianjin 300401, China.

## Abstract

The convergence of the Metaverse and the Internet of Things (IoT) paves the way for extensive data interaction between connected devices and digital twins; however, this simultaneously introduces considerable cybersecurity threats, including data breaches, ransomware, and device tampering. Existing intrusion detection algorithms struggle to effectively defend against emerging cyberattacks in the rapidly evolving Metaverse environment. Designing effective neural networks for intrusion detection algorithms relies heavily on expert experience, making the manual process time-consuming and often yielding suboptimal results. This paper addresses a critical gap in cybersecurity for Metaverse devices, which are often overlooked in traditional detection methods, and proposes an adaptive multiobjective evolutionary generative adversarial network (AME-GAN) as a novel, scalable solution for optimizing network intrusion detection. An inversely proportional hybrid attention-based long short-term memory GAN is proposed, combining GANs to generate minority class samples and alleviate the imbalance problem in training datasets, which has long hindered accurate intrusion detection. Additionally, an adaptive evolutionary neural architecture search algorithm for the supernet of the GAN is designed to guide the mutation direction of the supernet, enhancing the training stability. This paper further introduces a double mutation multiobjective evolutionary neural architecture search algorithm, integrating both the multiobjective evolutionary algorithm and the neural architecture search to optimize accuracy, real-time performance, and model diversity—a crucial aspect for Metaverse devices with diverse hardware constraints. Experiments conducted on 3 well-known datasets—NSL-KDD, UNSW-NB15, and CIC-IDS2017—demonstrate that AME-GAN outperforms state-of-the-art approaches, with improvements of 0.32% in accuracy, 0.31% in F1 score, 0.47% in precision, and 0.37% in recall. This paper offers a promising, adaptive framework to enhance cybersecurity in the Metaverse, improving detection performance and real-time applicability, and contributing to the future of network intrusion detection in next-generation digital environments.

## Introduction

In the Metaverse era, users leverage multi-device and multi-sensor collaboration to achieve multimodal data interactions involving text, audio, video, and motion from the real world, enabling entertainment and work in a virtual world that transcends reality [1]. The Metaverse incorporates cutting-edge technologies to offer personalized recommendations to consumers and deliver immersive and interactive experiences [2]. The advent of the Metaverse has introduced a new era of virtual reality, allowing individuals to immerse themselves in vast digital landscapes, interact in real time, and engage in activities ranging from gaming to socializing [3]. Integrating Internet of Things (IoT) such as sensor devices, wearables, and smart gadgets into the Metaverse environment is crucial for deepening interactions and enhancing immersion in the Metaverse. However, IoT devices typically employ minimal hardware designs, making them highly vulnerable to various types of network attacks and posing considerable security risks. Moreover, the Metaverse’s decentralized and distributed blockchain technology allows users to buy, sell, and trade virtual assets, making it an appealing target for attackers seeking to exploit vulnerabilities in smart contracts as well as steal non-fungible tokens (NFTs) and other valuable digital assets [4]. The openness and complex interactions within the Metaverse create potential cybersecurity risks, particularly threats related to network intrusions.

Network intrusion detection (NID) algorithms are designed to monitor and analyze network traffic, aiming to detect anomalous, intrusive, or noncompliant behaviors with precision and efficiency. NID algorithms are typically categorized into signature- and anomaly-based categories according to different working principles [5]. Signature-based NID algorithms rely on matching existing attack patterns, which are highly effective for known attacks but require extensive computational resources as the database grows and are ineffective for novel attacks [6]. Anomaly-based NID algorithms, on the other hand, detect abnormal activities by identifying deviations between user traffic and normal traffic, and perform better against novel attacks [7]. This kind of method can lead to a considerable number of false alarms. The pattern recognition scheme lays the foundation for enhancing NID algorithms through the efficient pattern learning capabilities of deep learning (DL) [8]. Various methods, including supervised learning, unsupervised learning, and reinforcement learning (RL), have been integrated into DL-based NID algorithms [9]. These NID algorithms rely heavily on feature extraction networks to distinguish between benign and malicious traffic. The widely recognized feature extraction networks are convolutional neural networks (CNNs) and Transformers. Transformer-based NID algorithms, as more recent developments, introduce the multi-head self-attention (MSA) mechanism to capture dependencies within input sequences and learn global–local relationships in a more comprehensive manner, which provides superior discriminative performance compared to CNN-based NID algorithms.

Although DL demonstrates its advantages in constructing hierarchical feature representations from labeled training data for analyzing and recognizing complex and high-dimensional Metaverse traffic [10], several challenges persist. Existing NID training datasets exhibit substantial disparities in sample distributions across categories, leading to a severe data imbalance issue. This imbalance results in substantial variations in detection accuracy across different types of threats in the Metaverse, ultimately reducing overall detection performance. Moreover, the development of NID models is largely dependent on expert prior knowledge, and the configuration of network layer parameters involves time-consuming manual adjustments through iterative training, which rarely results in the optimal architecture.

With the rapid expansion of the Metaverse driving a surge in data volume, there is a pressing need for an automated approach to develop efficient Metaverse NID algorithms that ensure precise detection and classification of diverse network traffic within the Metaverse while meeting real-time requirements. To address this challenge, we propose a Metaverse NID algorithm based on the adaptive multiobjective evolutionary generative adversarial network (AME-GAN). We utilize the powerful temporal feature extraction capability of long short-term memory (LSTM) networks and the attention mechanism’s proficiency in modeling global spatial features to extract spatiotemporal features from Metaverse traffic, thereby enhancing the performance of the original GAN. Moreover, the generator of the GAN is utilized to produce new rare-class samples, alleviating the data imbalance issue in the training datasets of Metaverse NIDs and contributing to improving the overall accuracy of the discriminator. By setting the discriminator’s accuracy and computational latency, as well as the generator’s fidelity and diversity, as optimization objectives in the GAN, an adaptive evolutionary search strategy is deployed within the supernet of the neural architecture search (NAS). This enables the automated search for discriminators with high accuracy and low latency, as well as generators with high fidelity and diversity.

### NID in the Metaverse

The ultimate goal of the Metaverse is to ensure that any event in the real world is instantly mirrored in the virtual world, while any changes within the Metaverse are accurately reflected in the physical world, achieving a true digital twin of both the real and virtual worlds [11]. To ensure that all users experience a secure and immersive environment within the Metaverse, there is an urgent need to establish flexible and cutting-edge security solutions capable of addressing the rapid transformations and dynamic developments inherent to the Metaverse. Artificial intelligence (AI)-driven machine learning-based NID network security solutions can effectively address a wide range of threats in network environments, providing more flexible and data-driven defense options for the Metaverse.

Machine learning-based NID algorithms automatically learn and adapt to new threats by analyzing data, making them ideal for dynamic and complex network environments. Deep learning, a subset of machine learning, models complex patterns through deep neural networks (DNNs), providing more powerful tools for NID [12]. DL-based NID algorithms have become a research hotspot, with CNN [13,14], LSTM [15], and Transformer [16,17] techniques substantially improving detection accuracy and reducing false positives [18]. CNN is particularly effective in capturing spatial patterns within network traffic [19]. The LSTM network demonstrates excellent performance in modeling temporal dependencies [20]. The work of [21,22] exploited CNN’s ability to capture spatial patterns in network traffic to construct a CNN-based NID algorithm that outperform traditional machine learning algorithms. However, CNN-based algorithms are not well-suited for capturing the long-term dependency of network traffic. In [20], bidirectional LSTM (BiLSTM) is proposed to solve intrusion detection problems, offering notable advantages over traditional LSTM in accuracy, precision, and F1 score on the NSL-KDD dataset. In [23], CNN and BiLSTM are combined to learn spatial and temporal features, exploring the effects of various hyperparameters and achieving better classification performance on the NSL-KDD and UNSW-NB15 datasets. Although LSTM alleviates the gradient vanishing problem to a certain extent, the problem still exists when dealing with very long sequences.

The self-attention mechanism in the Transformer can focus directly on the relationships between any 2 positions in a sequence, efficiently capturing complex global dependencies, making it highly effective for modeling long-distance dependencies [24]. CANET [25] integrates CNN and attention mechanisms, proposing a convolutional attention module to extract local spatiotemporal features. Additionally, it introduces an improved Equalization Loss v2, effectively enhancing the detection rate of minority classes and excelling other methods in detecting minority class attacks on the NSL-KDD and UNSW-NB15 datasets. ABD [26] enhances BiLSTM with an attention mechanism, improving its ability to capture contextual information and long-term dependencies in time-series data. This approach demonstrates stronger adaptability when handling complex network intrusion attacks, achieving 1.0% and 2.0% accuracy improvements in binary and multi-class tasks on the NSL-KDD dataset, respectively. The work of [27] abandons LSTM and proposes a temporal convolutional network (TCN) module, combining GAN and attention mechanisms to detect attacks. It outperforms existing GAN-based NIDs in both accuracy and speed, making it suitable for low-latency deployment on edge computing servers of IoTs. FlowTransformer [28] takes NetFlow-based data as input instead of packet-based data for monitoring, avoiding the privacy issues that packet capture may cause, and proposes a Transformer-based NID pipeline, which maintains the high accuracy and reduces the size of the encoder/decoder. The attention-based NID algorithms outperform the methods above in terms of detection accuracy. However, this improvement comes with a notable increase in model complexity, which is almost unbearable for Metaverse devices with minimal hardware design. Table 1 provides a comparison of the strengths and limitations of these methods.

**Table 1.** Comparison of NID in the Metaverse

Method	Strategy			Strengths	Weaknesses
	CNN	LSTM	Attention		
CNN-based NID [14–17]	√			Excellent at capturing local spatial patterns	Struggle with long-term dependencies
BiLSTM-based NID [18]		√		Long-term dependency support	Lacking the ability to extract local features
CNN + BiLSTM [19,20]	√	√		Handle local features and temporal dependencies	Ineffective with very long sequences and global contextual information
Transformer-based NID [21–23]			√	Stronger global modeling capability and long-term dependency capture ability	Computationally intensive and high tuning complexity
CANET [26]	√		√	Performance good at minority class attacks detecting	Overfitting on small datasets
ABD [27]		√	√	Combining attention and BiLSTM to effectively handle complex long-time sequences	High computational cost, unfriendly for low-computation devices
TCN [28]	√		√	Combining CNN, attention, and GAN to build low-latency models, good for IoT devices	Prone to mode collapse, leading to training instability
FlowTransformer [29]			√	Packet-based data reduces privacy concerns and outperformances at detection accuracy	Limited to NetFlow data and high model complexity

### GAN for NID

In addition to constructing neural network backbones with strong feature extraction capabilities for NID, numerous publicly available datasets for NID exhibit notable class imbalance, with benign instances vastly outnumbering malicious ones [29]. To this end, researchers have proposed methods to optimize training datasets based on Siamese neural networks (Siamese-NN) [30,31], extreme gradient boosting (XGBoost) [32], and generative models [33,34]. Generative models, especially GANs [35], have emerged as a prominent area of research because of their excellent diversity and authenticity in sample generation [36]. Each GAN consists of an adversarial structure consisting of a generator G and a discriminator D. The training process can be formulated as a minmax game $\underset{G}{\text{min}}\;\underset{D}{\text{max}}V\left(D,\;G\right)$. The objective of the discriminator is to maximize the probability of correctly classifying samples, identifying those that conform to the real data distribution $p_{\text{data}}$ as genuine, while rejecting as many fake samples generated by the generator as possible. The generator uses noise sampled from the $p_{n}$ distribution to generate samples, fitting the generated sample distribution to the real data distribution. This weakens the discriminator’s ability to identify real data and generated data, making it unable to effectively identify the generated data, thus producing incorrect classification results. During training, the generator and discriminator iteratively optimize against each other, ultimately reaching a Nash equilibrium where the generated data closely resemble real data.

$$\begin{array}{c} \underset{G}{\text{min}}\;\underset{D}{\text{max}}V(D,G)={𝔼}_{x∼p_{\text{data}}\left(x\right)}[\text{log}D\left(x\right)]\\ \;+{𝔼}_{n∼p_{n}\left(n\right)}[\text{log}\left(1-D\left(G\left(n\right)\right)\right)] \end{array}$$ (1)

GANs have been effectively applied to generate synthetic attack data, especially for challenging-to-obtain intrusion data. The diverse attack data generated by GANs can enhance the ability of NID to detect a wide range of threats [37]. Aydin [38] and Yang and Zhou [39] used the generator of GANs to create samples for dataset balancing by introducing a variational autoencoder to stabilize class distributions and learn the distribution of each class in the latent space. Higher-quality minority class samples can be generated. This effectively addresses the issues of insufficient sample diversity and mode collapse in traditional GANs, proving the feasibility of using GANs to solve the data imbalance issue. TMG-GAN [40] proposes a GAN-based data augmentation method with a multi-generator and classifier structure and introduces a classifier structure and incorporates cosine similarity computation to boost the quality of generated samples and strengthen class differentiation, addressing class imbalance in NID algorithms, achieving considerable detection performance improvement on CICIDS2017 and UNSW-NB15 datasets.

In practical NID applications, the generator of the GAN can generate data samples that simulate the characteristics of real network traffic [41]. In this context, algorithms that combine original GANs [42,43], DCGANs [44], WGANs [45,46], and other GAN variants to generate training data samples have been developed, addressing the problem of minority classes in training datasets. The work of [34,47–50] further proposed algorithms that use generators to generate malicious traffic, contributing to the development of more powerful and more adaptable NID algorithms. The internal discriminator demonstrates stronger adaptability in monitoring various new types of malicious traffic.

Hoang and Kim [51] proposed the convolutional adversarial autoencoder (CAAE) combined with an autoencoder and a GAN to learn the feature differences between normal and malicious traffic in a semisupervised manner, which substantially reduces both complexity and inference latency while ensuring robust detection performance, making it well-suited for real-time applications. Balaji and Narayanan [52] explored an alternative approach to reduce computational costs by using principal components analysis (PCA) for feature extraction and dimensionality reduction, followed by the application of BiGAN to detect anomalous network traffic. It greatly reduces the computational cost of the NID model, providing a faster and more efficient solution for network security. The comparative summary of the strengths and limitations of these methods is presented in Table 2.

**Table 2.** Comparison of GAN for NID

Method	Strengths	Weaknesses
GAN-based NID [39,40]	Combine the VAE and the GAN to generate high-quality minority class samples	VAE has insufficient capability to extract features for each class
TMG-GAN [41]	The multi-generator and classifier structure improves the quality of generated samples	The multi-generator and classifier structure increases the model complexity
GANs [42,43]	Generate minority class samples	Prone to mode collapse and hard to train the GAN model
DCGANs [44]	Enhance local feature learning and make the generated samples more realistic	The integration of multiple convolutional layers increases the computational cost
WGANs [46,47]	Wasserstein distance improves the training stability and enhances the accuracy and robustness of NID	The Wasserstein loss function and the backpropagation of the gradient penalty increase the computational complexity
Malicious traffic generation [34,48–51]	Generate malicious traffic samples to balance the dataset	Ethical concerns with malicious data
CAAE [52]	Combining CNN, autoencoder, and GAN and real-time detection supported	The complex network structure makes it unsuitable for large-scale datasets
BiGAN with PCA feature extraction [53]	Reduce dimensionality and speed up the training and detection process	PCA relies on high-quality training datasets and is unfriendly for imbalanced datasets

### NAS for GAN

To achieve better performance, the NID model design tends to be complex and deeper [53]. The neural network architecture design procedure relies more on the expertise [54]. Manually tuning the network parameters is a lengthy and repetitive process, making it challenging to identify the optimal settings [55]. Researchers have proposed realizing automated searches of the structural parameters of GANs via NAS. Three key elements make up NAS: the search space, search strategy, and performance estimation. The search process requires constructing a search space and then iteratively generating network architectures as candidates using a search strategy. Candidate architectures are evaluated with performance assessment methods, ultimately leading to the automatic selection of the optimal neural network design.

The early NAS methods were primarily developed based on RL. Methods such as AutoGAN [56] and AGAN [57] focused on employing the recurrent neural network (RNN) controller to steer the search process, accelerating the process via parameter sharing and dynamic resetting, and then rewarding the controller with the inception score (IS) of the neural network architecture, iteratively searching for the optimal structure. Unfortunately, these schemes are highly time consuming. To solve this issue and boost search efficiency, the work of [58–60] introduced the continuous relaxation (CR) search space to explore the convolutional cell structures of the generator and the discriminator in a differentiable way. The network architecture parameters are transformed into continuous variables, eliminating the need for brute-force searching of all discrete architectures. This allows the optimal GAN architecture to be efficiently found using gradient descent, considerably reducing time. CycleGANAS [61] conducted a more in-depth study by replacing the convolutional cell in AdversarialNAS [60] with a ResNet cell and employing single-level joint optimization to search for the neural network architecture and weights. This approach successfully stabilizes the search for a high-performance CycleGAN architecture, even in the presence of data imbalance.

Methods above have successfully automated the construction of generator and discriminator architectures; however, they do not address improvements to the adversarial mechanism of GANs. As a result, these methods are susceptible to local optima, exhibit poor training stability, and lack support for multiobjective optimization [62]. The work of [63] introduces evolutionary algorithms into NAS and employs many-to-one and one-to-one training strategies to optimize both the generator and discriminator, effectively improving the stability of GAN training and mitigating the issue of mode collapse. EAS-GAN [64] proposed an NAS strategy based on the evolutionary algorithm, integrating the generator evolution mechanism of evolutionary GAN [65] with NAS and sampling the population on the generator supernet to construct the population. The discriminator evaluates and retains the best offspring, thereby evolving a better architecture and weights. EGANS [9] further improves the above method by utilizing a collaborative dual evolution strategy under a unified evolutionary adversarial framework. It searches for the optimal generator and discriminator architectures, enhancing the adaptability and stability of GAN in zero-shot learning.

Generators and discriminators frequently encounter multiple objective functions that conflict with each other, making it impossible to merge them into a single objective problem. The method of selecting offspring from multiple variants based on evolutionary NAS improves the training stability of GANs yet leaves the multiobjective problem unresolved. MO-EGAN [66] formulates the training of GAN’s generator as a multiobjective optimization problem, optimizing 2 conflicting objectives: quality and diversity. It employs the Pareto dominance method for selection, improving the traditional EGAN and demonstrating outstanding performance in terms of generated sample quality and diversity. In [54], evolutionary weight-sharing GANs (EWSGAN) were introduced, advancing the field with a 2-stage NAS process where the first stage trains the supernet and the second stage applies multiobjective evolutionary NAS to find the optimal subnet. This method effectively reduces the search space and improves the search speed. The strengths and limitations of the respective methods are comparatively outlined in Table 3.

**Table 3.** Comparison of NAS for GAN

Method	Strategy			Strengths	Weaknesses
	RL	CR	Evolution		
AutoGAN [57], AGAN [58]	√			Utilize RNN controller for architecture search and parameter sharing	High computational cost and time consuming
Continuous relaxation search [59–61]		√		Use continuous relaxation to create differentiable search space and speed up the training	Still computationally intensive for complex GAN structures
CycleGANAS [62]		√		Employing single-level joint optimization to stabilize the search of architecture and weights	Complex structure brings higher computational cost
Evolutionary-based NAS [64]			√	Alleviate local optima and search for the optimal model architecture	Time consuming and hard to guarantee the detection speed
EGANS [9]			√	Improves training stability through cooperative coevolution	Requires more resources and time to train
MO-EGAN [66]			√	Multiobjective evolution to find the optimal generator structure and improve the quality and diversity of generated samples	Struggle with many objective optimization
EWSGAN [55]			√	Train the supernet and subnet in the 2-stage NAS process to reduce the search speed	Focuses only on the generator architecture search, neglecting the discriminator

### Motivations and contributions

We focus on generative artificial neural networks in the Metaverse, aiming to automate the construction of GAN-based Metaverse NID through NAS. This method utilizes the GAN’s generator to produce samples for minority classes, tackling the data imbalance issue in existing Metaverse NID datasets while aiming to strike an optimal balance between detection accuracy and real-time performance on Metaverse devices. The primary contributions of this study include:

a. To address the data imbalance issue in the Metaverse, we propose inversely proportional hybrid attention LSTM-GAN (IPHA LSTM-GAN) for feature extraction and minority class sample generation. LSTM is introduced to capture long-term dependencies in network traffic time series, whereas MSA is utilized to enhance the ability to extract global–local relationships. Furthermore, an inversely proportional generation (IPG) strategy based on cross-attention is adopted for noise-label fusion and minority class sample generation, improving the problem of data imbalance.
b. Unlike existing NAS algorithms, which focus on evolving architectures for sampled subnets, we introduce a novel adaptive evolutionary strategy to enhance the training stability in the Metaverse NID with GANs. Multiple mutation operators are applied simultaneously to the supernet, and through evaluation and selection, it retains the mutated supernet offspring architectures of the generator and discriminator with high accuracy. Automating the selection of evolutionary directions for the generator and discriminator supernets enhances the training stability of the Metaverse NID GAN.
c. To optimize multiple objectives in the Metaverse NID, a double mutation multiobjective evolutionary NAS algorithm is proposed. After applying multiple mutation operators to the supernet, the double mutation sampling (DMS) method combined with max, random, crossover, and mutation sampling strategies is used to obtain the subnet population, which helps to increase the diversity of the population. A multiobjective evaluation is then performed on the populations of generator and discriminator subnets, with nondominated solutions selected for cross-validation, thereby facilitating the search for the optimal architectures of the generator and discriminator within the GAN.

## Materials and Methods

**Fig. 1.**
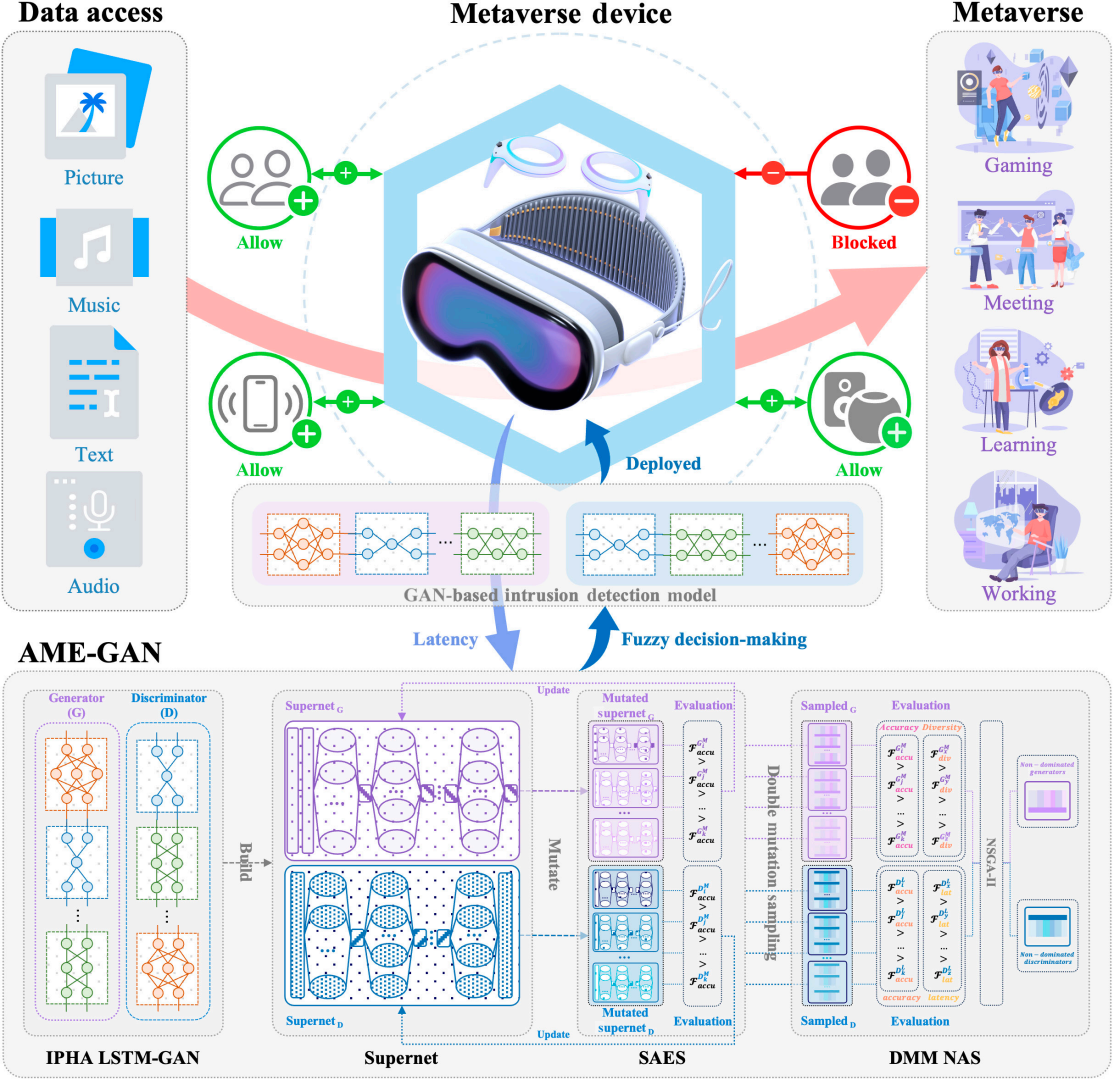
Overall framework of AME-GAN.

The overall framework of the proposed Metaverse intrusion detection algorithm is illustrated in Fig. 1. The algorithm deploys the candidate blocks of AME-GAN on the Metaverse device to measure the actual computational latency, which is fed back to AME-GAN for the multiobjective evolution of the generator supernet and the discriminator supernet. The automated search process then produces a GAN-based intrusion detection model comprising a generator and a discriminator. The model will be deployed on Metaverse devices to distinguish between normal and malicious traffic during data exchange with other devices, thereby blocking malicious access. As shown at the bottom of Fig. 1, the AME-GAN specifically consists of IPHA LSTM-GAN, a GAN-based supernet adaptive evolutionary strategy, and a double mutation multiobjective NAS (DMM NAS). IPHA LSTM-GAN is the intrusion detection model we aim to search for, which will be constructed as a generator supernet and a discriminator supernet. These 2 supernets will then mutate into multiple mutated supernets. The supernet adaptive evolutionary strategy (SAES) selects the best generator supernet and the best discriminator supernet based on their accuracy, stabilizing the GAN training process. DMM NAS employs DMS to sample both generators and discriminators and uses NSGA-II for multiobjective optimization to obtain nondominated models. Fuzzy decision-making is then used to construct the final intrusion detection model. The NSL-KDD dataset, UNSW-NB15 dataset, and CIC-IDS2017 dataset are then used to verify the performance of the algorithm.

### Inversely proportional hybrid attention LSTM-GAN

**Fig. 2.**
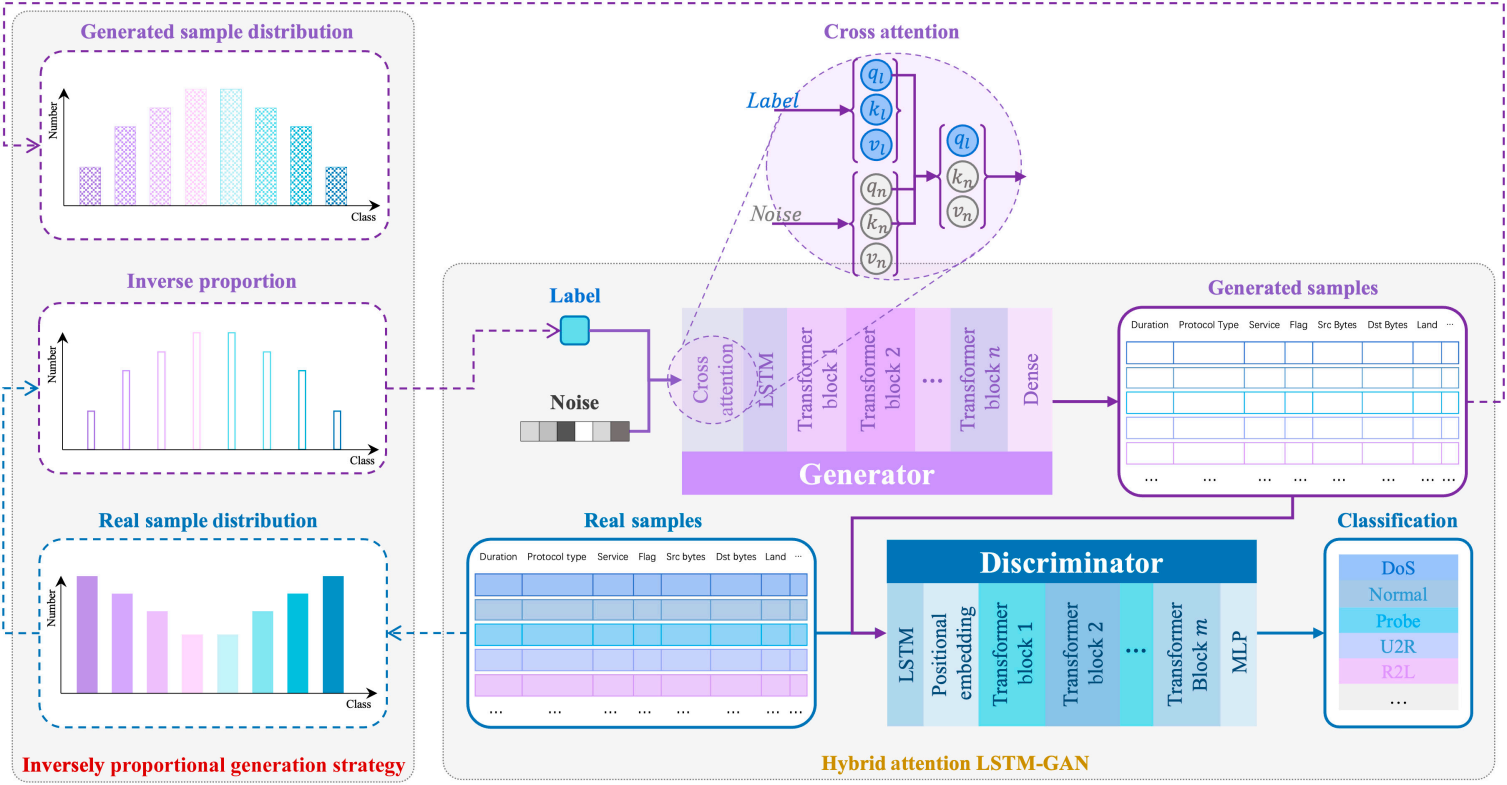
Inversely proportional hybrid attention LSTM-GAN.

The IPHA LSTM-GAN consists of 2 key components. As shown in Fig. 2, the first component is a hybrid attention LSTM-GAN (HA LSTM-GAN), which is used for feature extraction from network traffic sequences and specific class sample generation; the other component is an IPG strategy that increases the proportion of minority classes to balance the dataset. The combination of these 2 components helps to improve the low detection accuracy caused by the data imbalance issue.

#### Hybrid attention LSTM-GAN architecture

The overall structure of the HA LSTM-GAN is depicted in Fig. 2. Since the Metaverse NID takes network traffic time series as input for feature learning, and LSTM proves its effectiveness in modeling long-term dependencies of network traffic time series, we introduced BiLSTM into the generator and the discriminator of the GAN to compensate for the lack of temporal feature support in the original GAN. In terms of spatial features, HA LSTM-GAN adopts MSA of transformer block (TB) to strengthen global–local feature learning capabilities, enhancing the original GAN’s reliance on CNN’s local feature learning, and incorporating global temporal features. The combination of BiLSTM and MSA improves the spatiotemporal feature learning capability of the original GAN, leveraging the training stability as well.

The original GAN takes a normally distributed noise as input for sample generation. The generated sample categories are random and cannot be specified. For specific minority class sample generation, we construct the cross-attention mechanism (CAM) module to modify the generator architecture, so that the generator can take noise and labels as inputs, generating specific class samples, thereby avoiding the constraints of sample random generation of the generator and alleviating the dataset imbalance issue. The definition of the cross-attention function is calculated as follows:

$$\text{CrossAttention}(q_{l},k_{n},v_{n})=\text{Soft}\;\text{max}\left(\frac{q_{l}k_{n}^{T}}{\sqrt{d_{k}}}\right)v_{l}$$ (2)

where $q_{l}$ indicates the query of the input label. $k_{n}$ and $v_{n}$ indicate the key and value of the input noise. The CAM maintains the computational method of the original self-attention mechanism but replaces the query, key, and value from the same input with a different combination from different inputs. This method integrates multiple attention mechanisms, laying a theoretical foundation for the generation of specific class samples.

#### Inversely proportional generation

The training datasets of the Metaverse NID commonly suffer from the issue of data imbalance, and the synthetic minority oversampling technique (SMOTE) oversampling method is typically used to overcome the imbalance; however, the newly generated samples often resemble similar classes, leading to blurring of the class boundaries and reducing the detection efficiency. We propose the IPG method, as shown in Fig. 2. By calculating the inverse proportion of each class in the dataset, the generator generates samples based on this proportion, which increases the proportion of the minority classes and reduces that of major classes. The data imbalance issue is then improved. The IPG functions are described in Eqs. 3 and 4.

$$\sum_{1}^{n}p_{c_{i}}=1,i\in \{1,2,\cdots ,n\}$$ (3)

$$p_{c_{i}}^{\prime }=\frac{\text{ln}\frac{1}{p_{c_{i}}}}{\sum_{1}^{n}\text{ln}\frac{1}{p_{c_{i}}}},i\in \{1,2,\cdots ,n\}$$ (4)

where $c_{i}$ denotes the ith class of the dataset, n denotes the number of classes, $p_{c_{i}}$ indicates the proportion of $c_{i}$ in the dataset, and $p_{c_{i}}^{\prime }$ indicates the inverse proportion of $c_{i}$.

The IPG guides the GAN’s generator to produce more samples from the minority class and fewer from the majority class, enriching the feature learning set for the discriminator and helping to address the low detection accuracy caused by the data imbalance in the original dataset.

### GAN-based supernet adaptive evolutionary strategy

#### Search space and supernet

The NAS we proposed follows the same idea of CR as in FBNet [59] to construct a search space for TB structure parameters. This allows us to update both the candidate block weights and layer parameters through backpropagation in a differentiable way and explore the generator and discriminator architectures of the HA LSTM-GAN with high efficiency. As depicted in Fig. 2, the generator and the discriminator contain $l_{n}$ and $l_{m}$ TBs, respectively. Notably, the numbers of $l_{n}$ and $l_{m}$ do not need to be consistent.

In the process of architecture searching, exploring as many hyperparameter combinations as possible is the better solution, but more combinations make the search space size of the supernet grow exponentially and substantially increase the search time. The performance gain for the final architecture is also not as obvious as expected. Considering the computational limitations of devices, we aim to search for the lightweighted neural networks. The search focuses on the head and feedforward dimension (ff-dim) parameters within the TB structure. The values for these parameters are [4, 8, 16] for the head and [32, 64, 128, 256, 512] for ff-dim, resulting in a total of 15 candidate blocks. In addition, to reduce the unnecessary TBs in the generator and discriminator, a skip operator is added starting from the second TB, allowing the model to skip the current TB while ensuring that at least one TB is included for feature extraction. We construct a supernet for both the generator and the discriminator via these 16 candidate blocks, forming a search space of size $15^{2}\times 16^{l_{n}+l_{m}-2}$. The detailed TB parameters are shown in Table 4.

**Table 4.** Candidate blocks

ID	Transformer block	Head	ff-dim	ID	Transformer block	Head	ff-dim
1	h4_f32	4	32	9	h8_f256	8	256
2	h4_f64	4	64	10	h8_f512	8	512
3	h4_f128	4	128	11	h16_f32	16	32
4	h4_f256	4	256	12	h16_f64	16	64
5	h4_f512	4	512	13	h16_f128	16	128
6	h8_f32	8	32	14	h16_f256	16	256
7	h8_f64	8	64	15	h16_f512	16	512
8	h8_f128	8	128	16	Skip	-	-

#### Supernet adaptive evolutionary strategy

**Fig. 3.**
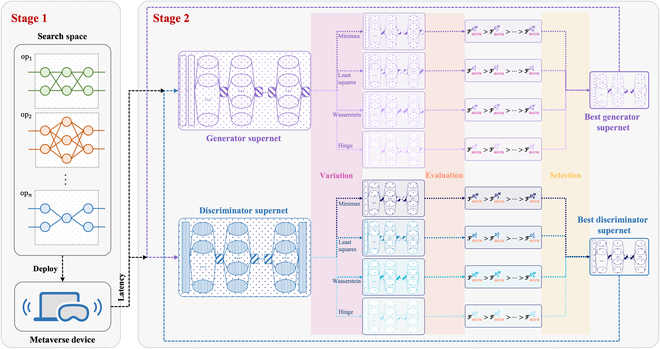
GAN-based adaptive evolutionary strategy for supernet.

Existing NAS algorithms typically evolve sampled subnets. We evolve not only the sampled subnet but also the supernet by introducing SAES based on GANs. As depicted in Fig. 3 (stage 2), our strategy utilizes minimax, least-squares, Wasserstein, and hinge loss operators to mutate the supernet and generate offspring. The mutation of the supernet is driven by the loss function, which is computed by calculating the generator and discriminator supernet losses and performing gradient backpropagation. This paper introduces 4 mutation operators, allowing the generator and discriminator supernets to each produce 4 mutated offsprings. The specific loss functions for these operators are detailed below. Subsequently, the offspring are evaluated by calculating the generation accuracy of the generator offsprings and the training accuracy of the discriminator offsprings. The generation and training accuracy is then sorted respectively, with the top-performing model selected. The mutation directions for both the generator and discriminator supernets are automatically determined, resulting in more robust and stable GAN training.

a. Minimax mutation: This mutation corresponds to the minimax objective function in the original GAN [35], where the loss function defines a zero-sum game between the generator G and discriminator D. The generator produces samples to fit the real data from a specific class, while the discriminator seeks to identify both the authentic samples and generated samples. Unfortunately, the generator often produces samples that are concentrated in a few modes under this mutation, leading to mode collapse. The objective functions are defined as follows:
  $$L_{D}^{\text{Minimax}}={𝔼}_{x∼p_{\text{data}}\left(x\right)}\left[\text{log}D\left(x\right)\right]+{𝔼}_{n∼p_{n}(n)}[\text{log}\left(1-D\left(G\left(n\right)\right)\right)]$$ (5)
  $$L_{G}^{\text{Minimax}}={𝔼}_{n∼p_{n}\left(n\right)}[\text{log}\left(1-D\left(G\left(n\right)\right)\right)]$$ (6)
  Here, D(x) denotes the discriminator’s output for the real sample x, while G(n) denotes the new sample generated by the generator from the random noise *n*. ${𝔼}_{x∼p_{\text{data}}\left(x\right)}$ refers to the expectation of data sampled from the real data distribution $p_{\text{data}}$, while ${𝔼}_{n∼p_{n}\left(n\right)}$ refers to that from the noise distribution $p_{n}$. As D improves its ability to distinguish, it is easier to identify the samples G(n) generated by G and the value of $D\left(G\left(n\right)\right)$ approaches 0. Consequently, the cross-entropy objective function $L_{D}^{\text{Minimax}}$ of D progressively increases. In contrast, as G improves its generation ability, it becomes more difficult to distinguish the new sample and the value of $D\left(G\left(n\right)\right)$ approaches 1, and the objective function $L_{G}^{\text{Minimax}}$ of G decreases.
b. Least-squares mutation: Least-squares mutation is derived from least-squares GAN (LSGAN) [67], which uses the least-squares objective function ${\left(D\left(x\right)-n_{\text{real}}\right)}^{2}$ to provide larger gradients. This helps alleviate the vanishing gradient problem that occurs when D(G(n) approaches 0 during minimax mutation in the original GAN. By doing so, it resolves the issue of generator weight updates, reduces the occurrence of mode collapse, and accelerates the training process. The objective functions are defined as follows:
  $$\begin{array}{c} L_{G}^{\text{Least}-\text{Square}}=\\ {𝔼}_{x∼p_{\text{data}}\left(x\right)}[{\left(D\left(x\right)-N_{\text{real}}\right)}^{2}]+{𝔼}_{n∼p_{n}\left(n\right)}[{\left(D\left(G\left(n\right)\right)-N_{new}\right)}^{2}] \end{array}$$ (7)
  $$L_{G}^{\text{Least}-\text{Square}}={𝔼}_{n∼p_{n}\left(n\right)}\left[{\left(D\left(G\left(n\right)\right)-N_{\text{encoding}}\right)}^{2}\right]$$ (8)
  where $N_{\text{real}}$ and $N_{new}$ denote the class encoding numbers of the real sample and the new sample, respectively, and $N_{\text{encoding}}$ denotes the encoding number of the sample that the generator is expected to produce.
c. Wasserstein mutation: Originating from the Wasserstein GAN (WGAN) [68], this mutation assesses the discrepancy between the distributions of real and generated data using Wasserstein distance. This approach effectively addresses the limitation in the original GAN, where the nonoverlapping distributions between real and generated data cause the Jensen–Shannon divergence to lead to gradient vanishing, hindering the backpropagation and weight updates in the original GAN. The objective functions are defined as follows:
  $$L_{D}^{\text{Wasserstein}}={𝔼}_{x∼p_{\text{data}}\left(x\right)}\left[D\left(x\right)\right]+{𝔼}_{n∼p_{n}\left(n\right)}\left[D\left(G\left(n\right)\right)\right]$$ (9)
  $$L_{G}^{\text{Wasserstein}}={𝔼}_{z∼p_{z}\left(z\right)}\left[D\left(G\left(z\right)\right)\right]$$ (10)
d. Hinge mutation: By combining the hinge loss with the original GAN [69], this mutation can provide stable gradient updates and a training process by constraining the growth of the objective function. The discriminator’s objective value exists when the discriminator output D(x) for a real sample is less than 1 and that for the generated sample $G\left(n\right)$ is greater than −1. Otherwise, the value of the objective function equals 0, causing the gradient to vanish. The objective functions are described as follows:
  $$\begin{array}{c} L_{G}^{\text{Hinge}}={𝔼}_{x∼p_{\text{data}}(x)}[\text{max}\left(0,\;1-D\left(x\right)\right)]\\ \;+{𝔼}_{n∼p_{n}(n)}[\text{max}\left(0,\;1+D\left(G\left(n\right)\right)\right)] \end{array}$$ (11)
  $$L_{G}^{\text{Hinge}}={𝔼}_{n∼p_{n}(n)}[D\left(G\left(n\right)\right)]$$ (12)

SAES is mainly used in stage 2 (model pretraining) of the NAS framework to improve the stability of the subsequent search process and accelerate the convergence speed. In this process, only the layer parameters are trained in this process, with no updates to the candidate block weights. In the process, the discriminator supernet takes the generated samples of the randomly initialized generator supernet as inputs. The discriminator supernet is then duplicated into multiple copies, each of which is mutated by the mutation operator mentioned above to generate the supernet offspring. We evaluate the detection accuracy of all mutated offsprings, keeping the highest one for the next iteration. The generator supernet uses the same method to obtain offspring. These offspring are evaluated with the same noise to generate samples, and their generative accuracy is calculated using the previously retained discriminator, keeping the highest one for the next iteration.

### Double mutation multiobjective NAS

#### Optimization objectives

The Metaverse NID we proposed has 3 purposes: improving dataset imbalance, searching for light-weighted neural network architectures, and enhancing the detection accuracy, which means that the Metaverse NID is not a single-objective problem and that multiple optimization objectives need to be set. Since the discriminator of the GAN is responsible for identifying the normal and malicious traffic in the Metaverse NID, it is important to set the detection accuracy as one of the optimization objectives. Moreover, non-real-time Metaverse NID makes no sense for Metaverse devices, and the actual computational latency of Metaverse devices is often ignored; hence, the computational latency is also required as an optimization objective. While the generator in GANs excels at generating accurate samples, batch production often results in similar samples, limiting the diversity of newly generated data and hindering the practical use of this technology. Therefore, we optimize the NAS process with the following objectives: detection accuracy and computational latency of the discriminator, and generative accuracy and diversity of the generator. The discriminator and generator multiobjective optimization problems can be described as follows:

$$\begin{array}{c} \text{max}\;F_{Dis}\left(x\right)={\left[f_{Dis_{\text{Accu}}}\left(x\right),\;f_{Dis_{Lat}}\left(x\right)\right]}^{T}\\ \text{subject to:}\;0\leq f_{Dis_{\text{Accu}}}\left(x\right)\leq 1,f_{Dis_{Lat}}\left(x\right)>0,x\in X \end{array}$$ (13)

$$\begin{array}{c} \text{max}\;F_{Gen}\left(x\right)={\left[f_{Gen_{\text{Accu}}}\left(x\right),\;f_{Gen_{Div}}\left(x\right)\right]}^{T}\\ \text{subject to:}\;0\leq f_{Gen_{\text{Accu}}}\left(x\right)\leq 1,f_{Gen_{Div}}\left(x\right)>0,x\in X \end{array}$$ (14)

where the detection accuracy $f_{Dis_{\text{Accu}}}(x)$ and the generative accuracy $f_{Gen_{\text{Accu}}}(x)$ are obtained by feeding x into the discriminator of the IPHA LSTM-GAN. The computational latency $f_{Dis_{Lat}}\left(x\right)$ is calculated by accumulating the average computational latency of each candidate block. The average computational latency is acquired by deploying the candidate blocks on the target Metaverse device and measuring the runtime over multiple runs. The diversity $f_{Gen_{Div}}\left(x\right)$ is improved by IS, which achieves the goal of minimizing the increase in computational cost. The latency and diversity function can be described as follows:

$$f_{Dis_{Lat}}\left(x\right)=\frac{1}{t_{Lat}}$$ (15)

$$f_{Gen_{Div}}(x)=\frac{\text{exp}\left(\frac{1}{N},\;\sum_{i=1}^{N},\;D_{KL},\;\left(p\left(y|x_{i}\right)\;\|p\left(y\right)\right)\right.}{\frac{1}{N}\sum_{i=1}^{N}{\left(PE\left(x_{i}\right)-PE\left(x_{\text{real}}\right)\right)}^{2}}$$ (16)

where $t_{Lat}$ denotes the average computational latency. $x_{i}$ denotes the ith generated sample, and $x_{\text{real}}$ denotes the real sample. N denotes the batch size, and $p\left(y,\;x_{i}\right)$ represents the conditional class distribution obtained by the discriminator after classifying the generated sample $x_{i}$, p(y) denotes the marginal class distribution of all generated samples, $PE\left(x_{i}\right)$ denotes the relative positional encoding vector of $x_{i}$, and $D_{KL}\left(p\left(y|x_{i}\right)\|p\left(y\right)\right)$ calculates the Kullback–Leibler (KL) divergence between the conditional class distribution $p\left(y\;,\;x_{i}\right)$ of the generated sample $x_{i}$ and the marginal distribution p(y). The generator diversity function enhances the reliability of diversity evaluation by introducing the relative positional distance between real and generated samples.

#### Double mutation NAS

**Fig. 4.**
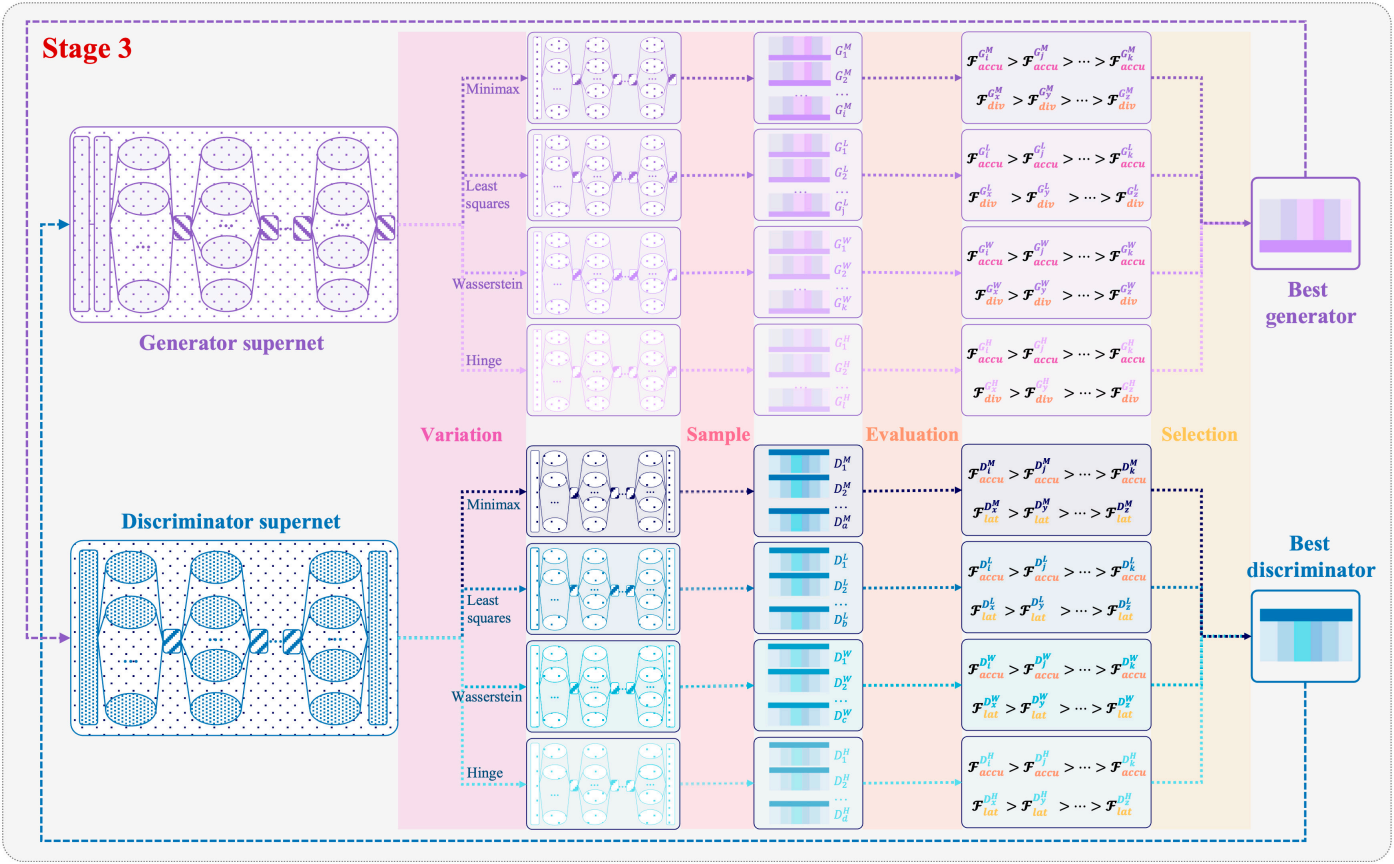
Double mutation multiobjective NAS.

Figure 4 illustrates the DMS-based double mutation NAS (DM NAS) algorithm, which is primarily applied in stage 3 for weight training. The training method in this stage is very similar to that in stage 2. The difference is that a sampling operation is added after supernet mutation. This enables the construction of a subnet population by applying the DMS. The mutated supernet with the largest nondominated solution set is retained as the optimal supernet after multiobjective evaluation and selection. Moreover, the generator and discriminator subnets with the highest generative/detection accuracy are also obtained in this process, which are used for the cross-validation of the generator and discriminator subnets in the next iteration. Through repeated iterations, the nondominated sets of discriminator and generator subnets are eventually obtained. A fuzzy decision-making method, using the triangular membership function and weighted average technique, is applied to select the best discriminator and generator from the nondominated sets. The GAN architecture is then searched. Fine-tuning the optimized GAN model produces a final model that can be deployed on Metaverse devices.

We utilize the CR of candidate blocks for NAS, aiming to search for the optimal candidate block with the highest weight. By sampling each layer’s optimal candidate block through max sampling, the generator and discriminator subnets are constructed. However, weight maximization does not always reliably select candidate modules that accurately reflect the optimal performance when the network has not converged. In this regard, we sample the mutated generator and discriminator supernet with a DMS that combines multiple sampling methods of max sampling, random sampling, crossover sampling, and mutation sampling.

a. Max sampling: Selects the candidate blocks with the highest weight in each layer.
b. Random sampling: Randomly selects any candidate block in the layer.
c. Crossover sampling: Crosses over a gene segment between positions a and b (where a>b) from the gene sequences of any 2 optimal subnets in the previous iteration to generate 2 new subnets.
d. Mutation sampling: Mutate a gene at any position within the gene sequence of one optimal subnet in the previous iteration to form a new subnet.

Algorithm 1 provides the pseudocode for the DMS. When training enters the stage 3 (weight training phase), subnet sampling begins. First, one subnet is sampled via the max sampling method. Then, additional subnets are sampled to build the population by randomly choosing one of the following 2 schemes: (a) select any 2 individuals from the nondominated solution set in the previous iteration, perform crossover sampling to generate 2 new individuals, and then use the random sampling to sample N−3 additional individuals; (b) select any one individual from the nondominated solution set in the previous iteration, generate a new individual via the mutation sampling, and then use the random sampling method to sample N−2 additional individuals. In this way, the population with new individuals is constructed in each iteration.


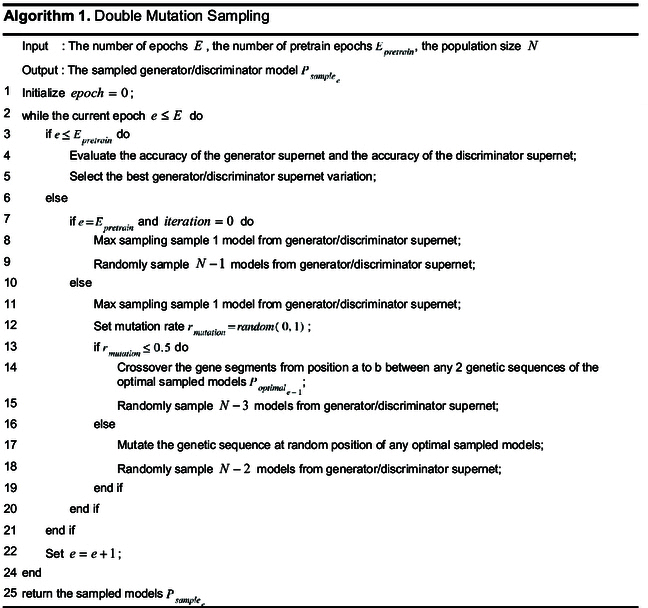


### Datasets

The experiments were conducted on the NSL-KDD, UNSW-NB15 [70], and CIC-IDS2017 datasets, where the issue of data imbalance is prominent in all 3.

#### NSL-KDD dataset

The NSL-KDD dataset, an enhanced version of the KDD CUP 99 dataset, is specifically designed for NID research. While the KDD CUP 99 dataset is based on the DARPA98 network traffic data, it has faced considerable criticism due to issues such as data redundancy and imbalance, which hinder the effectiveness of NID research. The NSL-KDD dataset reduces the amount of duplicated data and balances the samples, enhancing the effectiveness and consistency of model evaluation and making itself an essential benchmark for NID algorithms. The dataset has 41 features, with the training subset containing 22 known attack types and 17 more unknown attack types than the test subset. All the data are categorized into 5 main classes. Detailed information about the NSL-KDD is shown in Table 5.

#### UNSW-NB15 dataset

Developed by the Australian Centre for Cyber Security, the UNSW-NB15 dataset offers a comprehensive collection of normal traffic and various attack types from modern network environments, making it an essential benchmark for NID research. The dataset is generated by the IXIA PerfectStorm tool, contains 42 features, and covers 10 main classes. The detailed information about the UNSW-NB15 dataset is shown in Table 5.

#### CIC-IDS2017 dataset

The CIC-IDS2017 dataset, developed in collaboration between the Communications Security Establishment (CSE) and the Canadian Institute for Cybersecurity (CIC), serves as a valuable resource for NID research. It contains a collection of contemporary attack scenarios based on real-world network traffic data. It has 79 features and covers 15 main classes. The detailed information about the CIC-IDS2017 dataset is shown in Table 5.

**Table 5.** Details of NSL-KDD, UNSW-NB15, and CIC-IDS2017 datasets

Dataset	Feature number	Class number	Types of attacks	Train		Test	
				Number	Rate (%)	Number	Rate (%)
NSL-KDD	41	5	Normal	67,343	53.46	9,711	43.08
			Dos	45,927	36.46	7,548	33.48
			Probe	11,656	9.25	2,421	10.74
			R2L	995	0.79	2,754	12.22
			U2R	52	0.04	200	0.89
			Total	125,973	100.00	22,544	100.00
UNSW-NB15	42	10	Normal	56,000	31.94	37,000	44.94
			Fuzzers	18,184	10.37	6,062	7.36
			Analysis	2,000	1.14	677	0.82
			Backdoors	1,746	1.00	583	0.71
			DoS	12,264	6.99	4,089	4.97
			Exploits	33,393	19.04	11,132	13.52
			Generic	40,000	22.81	18,871	22.92
			Reconnaissance	10,491	5.98	3,496	4.25
			Shellcode	1,133	0.65	378	0.46
			Worms	130	0.07	44	0.05
			Total	175,341	100.00	82,332	100.00
CIC-IDS2017	79	15	BENIGN	1,817,056	80.3189	454,264	80.3189
			DoS Hulk	184,099	8.1377	46,025	8.1377
			PortScan	127,043	5.6157	31,761	5.6157
			DDoS	102,420	4.5272	25,605	4.5272
			DoS GoldenEye	8,234	0.3640	2,059	0.3640
			FTP-Patator	6,348	0.2806	1,587	0.2806
			SSH-Patator	4,718	0.2085	1,179	0.2085
			DoS slowloris	4,637	0.2050	1,159	0.2050
			DoS Slowhttptest	4,399	0.1945	1,100	0.1945
			Bot	1,565	0.0692	391	0.0692
			Web Attack—Brute Force	1,206	0.0533	301	0.0533
			Web Attack—XSS	522	0.0231	130	0.0231
			Web Attack—Sql Injection	17	0.0007	4	0.0007
			Infiltration	29	0.0013	7	0.0013
			Heartbleed	9	0.0004	2	0.0004
			Total	2,262,301	100.0000	565,575	100.0000

### Implementation settings

#### Implementation platform

The hardware platform used in the experiments of this paper contains an Intel Xeon(R) Platinum 8352V CPU, 40 GB of RAM, and an Nvidia GeForce RTX 3090 discrete graphics card with 24 GB of VRAM. The software platform is based on the 64-bit Ubuntu 20.04.6 LTS operating system.

#### Dataset preprocessing

The datasets mentioned above provide data types that include integer features, float features, Boolean features, and categorical features. Since the neural network of the Metaverse NID cannot handle the data with a variety of data types, all the data must be transferred into the float32 data type to be compatible with the DNN frameworks.

To this end, the dataset preprocessing begins with data cleaning. Columns with all 0s are regarded as irrelevant features, and rows containing NaN or INF are considered as error data. Dropping all these data cuts down the amount of training data. Later, the text data of categorical features are converted into numeric data via one-hot encoding. Although it increases the number of features, this method converts each category into an independent binary vector, enabling the machine learning model to correctly handle categorical data and avoid misinterpreting the relationships between categories. All numeric data are then normalized to the range of [0, 1] using min-max normalization, ensuring that features with larger ranges do not dominate the learning process, thus avoiding overfitting and improving the convergence speed during model training. Furthermore, the NSL-KDD and UNSW-NB15 datasets come pre-split into training and testing subsets, with each subset stored in its own file. We use the training subset for model training and the remaining portion for validation. However, CIC-IDS2017 consists of 7 network traffic files and does not provide a predefined split between the training set and the test set. To expedite model performance evaluation on this large-scale dataset, we randomly select 1% of the data as the test subset, with the remaining 99% used for training.

## Results

### Evaluation metrics

We evaluate the performance of the proposed algorithm using accuracy (ACC), precision (PRC), recall (RCL), and F1 score (F1), which are standard metrics in NID algorithms. These metrics are based on false negatives (FN), false positives (FP), true positives (TP), and true negatives (TN), which can be calculated as follows:

$$\text{Accuracy}=\frac{TP+TN}{TP+TN+FP+FN}$$ (17)

$$\text{Precision}=\frac{TP}{TP+FP}$$ (18)

$$\text{Recall}=\frac{TP}{TP+FN}$$ (19)

$$F1-\text{score}=\frac{\text{Precision}\times \text{Recall}}{\text{Precision}+\text{Recall}}\times 2$$ (20)

### Comparison with state-of-the-art methods

This section aims to compare our approach with other NID algorithms. However, we have not yet determined the exact number of TB to search for in the model. While adding more TB modules theoretically benefits model performance, the high computational cost of the attention mechanism implies that stacking excessive TBs results in a substantial expansion of the NAS search space, severely affecting the algorithm’s convergence speed. Furthermore, adding more TB modules in the later stages results in diminishing returns in terms of performance improvement. To address this, we conducted experiments on each dataset using a multi-layer stacking method to identify the optimal number of TB modules for each dataset.

Table 6 displays the accuracy results. An increase in the number of TB layers does not lead to a continuous improvement in accuracy. The accuracy of NSL-KDD and UNSW-NB15 stops increasing when the TB layer reaches 5. In the CIC-IDS2017 dataset, accuracy shows a slight decline at 4 network layers and a notable decrease at 6 layers. Due to the presence of the skip option in the TB candidate blocks, a larger TB layer number allows NAS to reduce redundant computation through skipping. Therefore, to ensure that the network achieves its maximum performance, we increased the number of layers from the optimal configuration and set the number of TB layers to 6.

**Table 6.** Accuracy comparison with different transformer block layer numbers on NSL-KDD, UNSW-NB15, and CIC-IDS2017 datasets

		Transformer block layer number					
	Dataset	1	2	3	4	5	6
Accuracy	NSL-KDD	74.00%	74.14%	76.02%	76.43%	76.71%	75.89%
	UNSW-NB15	73.44%	73.97%	73.65%	74.15%	74.84%	71.28%
	CIC-IDS2017	92.63%	95.82%	96.22%	96.78%	96.38%	94.32%

After determining the number of TBs, we compare our method with a variety of NID algorithms based on typical and multiobjective evolutionary schemes. All the algorithms are trained and tested on the NSL-KDD, UNSW-NB15, and CIC-IDS2017 datasets. The typical NID algorithms include representative models such as CNN-BiLSTM [19], BiDLSTM [18], BiLSTM-DNN [27], ABD [27], CANet [26], and FlowTransformer [29] in recent years. The multiobjective evolutionary intrusion detection algorithms include MECNN [71], EvoBMF [72], and MENAS [73]. All the neural network models were retrained based on the corresponding dataset to ensure fairness in comparison.

We use the training subsets of all these datasets for the training of the Metaverse NID models and validate the models on the test subsets. Table 7 shows the results of all evaluation metrics of all the algorithms including the competitors and our algorithm. As shown in the table, our method is superior in almost all the evaluation metrics mentioned above. The detection accuracies reach 79.17%, 77.97%, and 99.75%. The typical NID algorithms mainly utilize the combinations of CNNs, BiLSTM, and Transformers to enhance model performance, which we also utilize. Additionally, we propose DM NAS, and the results prove its positive impact on enhancing the performance of the Metaverse NID. Compared with the multiobjective evolution-based NID algorithms, all algorithms including ours use the same fast nondominated sorting to select the optimal solutions in multiobjective optimization. However, the competitors search for the CNN-only neural network models, which lack the spatiotemporal feature learning capabilities provided by BiLSTM and Transformer. The result shows the advantages of our HA LSTM-GAN with BiLSTM and Transformer introduced. Furthermore‌, the advantage is evident on all datasets, indicating that our method possesses a certain degree of generalization capability.

**Table 7.** Multi-class classification performance comparisons with state-of-the-art algorithms in terms of 4 evaluation metrics on NSL-KDD, UNSW-NB15, and CIC-IDS2017 datasets. Note that the higher value of all 4 metrics is better.

Scenario		NSL-KDD				UNSW-NB15				CIC-IDS2017			
Method	Year	ACC (%) ↑	F1 (%) ↑	PRC (%) ↑	RCL (%) ↑	ACC (%) ↑	F1 (%) ↑	PRC (%) ↑	RCL (%) ↑	ACC (%) ↑	F1 (%) ↑	PRC (%) ↑	RCL (%) ↑
CNN-BiLSTM [19]	2020	76.45	72.46	72.92	76.45	77.03	76.39	78.53	77.03	99.49	99.69	99.89	99.49
BiDLSTM [18]	2021	76.96	72.69	78.35	76.96	68.74	69.38	76.61	68.74	99.35	99.52	99.69	99.35
BiLSTM-DNN [27]	2023	78.72	75.19	81.41	78.72	71.21	66.85	64.09	71.21	99.60	33.27	99.37	33.31
ABD [27]	2023	73.35	43.88	45.47	44.70	69.76	70.54	78.17	69.76	99.57	40.78	99.77	39.25
CANet [26]	2023	77.68	53.41	47.98	49.84	75.83	75.22	78.75	75.83	99.49	99.68	99.89	99.49
FlowTransformer [29]	2024	78.16	74.92	78.66	78.16	74.86	75.09	78.66	74.68	99.59	99.77	99.95	99.59
MECNN [71]	2022	74.73	50.22	76.82	74.73	75.70	75.08	78.43	75.70	99.24	61.54	71.62	59.12
EvoBMF [72]	2023	74.04	48.72	66.87	74.04	77.48	76.48	78.35	77.48	99.67	68.83	75.43	66.43
MENAS [73]	2024	77.52	54.37	80.64	77.52	43.01	43.27	44.79	43.01	99.73	77.60	82.97	76.16
Proposed	2024	79.17	75.66	82.40	79.17	77.97	76.84	79.15	77.97	99.75	99.86	99.98	99.75

### Model diversity comparison

**Fig. 5.**
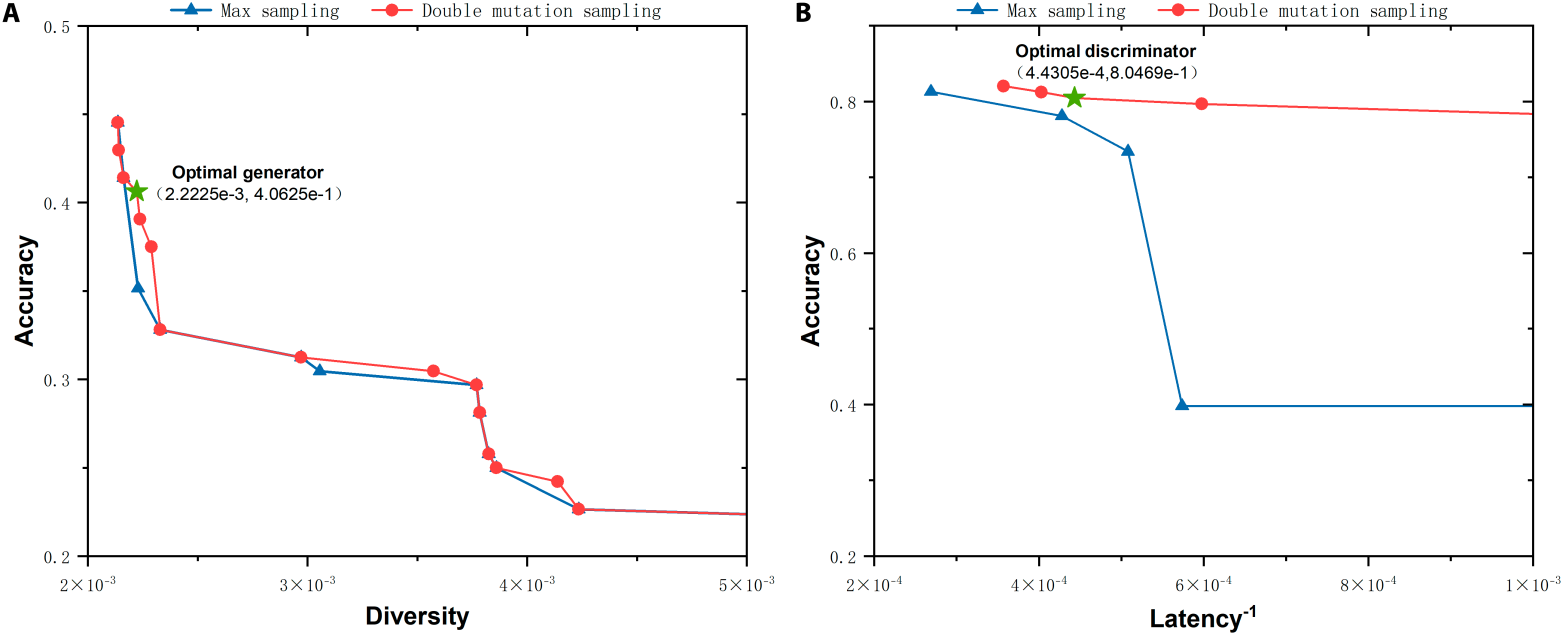
Max sampling and DMS nondominated set comparison on the NSL-KDD dataset. (A) Diversity–accuracy of generator subnets. (B) Latency–accuracy scatter of discriminator subnets. Green star represents the optimal generator/discriminator selected by fuzzy decision-making method.

**Fig. 6.**
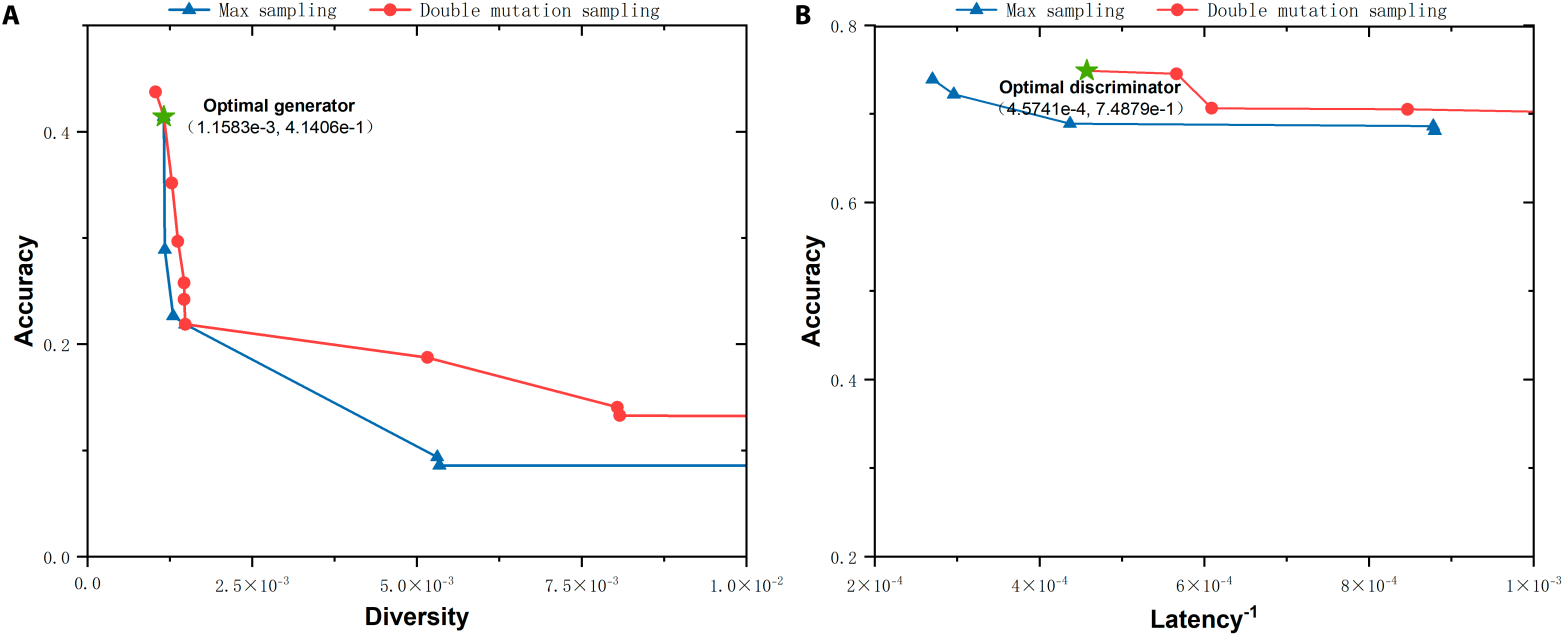
Max sampling and DMS nondominated set comparison on the UNSW-NB15 dataset. (A) Diversity–accuracy scatter of generator subnets. (B) Latency–accuracy scatter of discriminator subnets. Green star represents the optimal generator/discriminator selected by fuzzy decision-making method.

**Fig. 7.**
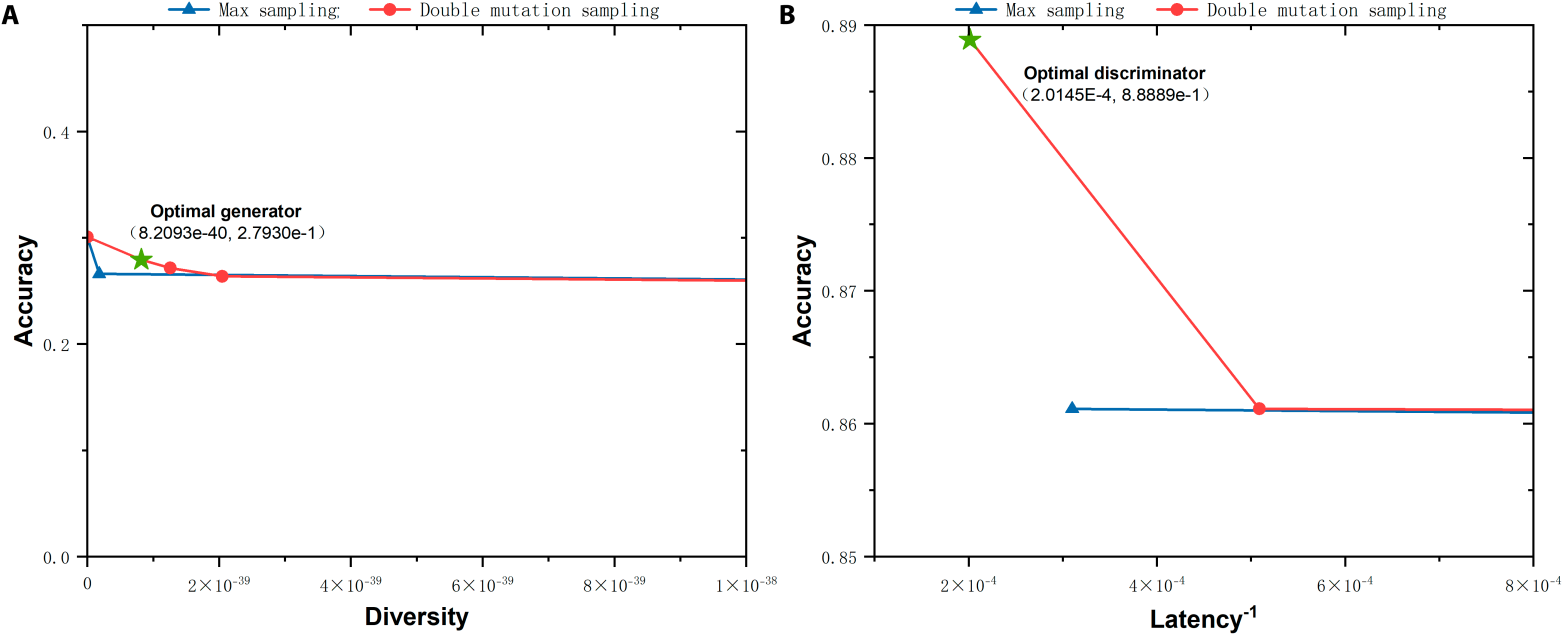
Max sampling and DMS nondominated set comparison on the CIC-IDS2017 dataset. (A) Diversity–accuracy scatter of generator subnets. (B) Latency–accuracy scatter of discriminator subnets. Green star represents the optimal generator/discriminator selected by fuzzy decision-making method.

To determine how the DMS method enhances model diversity compared with max sampling, we provide max sampling results and DMS results from the multi-class classification experiments on the datasets mentioned above. The results are shown in Figs. 5 to 7. All figures contain subplots A and B, which are nondominated set plots of the optimization objectives for the max sampling and DMS. Notably, the vertical and horizontal axes represent the sample generative accuracy and diversity of the generator in subplot A, whereas in subplot B, the axes represent the detection accuracy of the discriminator and the reciprocal of the discriminator computational latency, respectively. Higher values of these metrics indicate better performance; thus, the upper-right points indicate superior overall performance. The upper-right DMS points consistently occupy a dominant position compared to the max sampling points. To provide a more intuitive comparison of the performance between the max sampling and DMS methods, we calculate the hypervolume (HV) metric for the nondominated solution sets generated by these 2 approaches. Table 8 shows that the proposed DMS sampling method results in higher HV values for these datasets. The findings confirm that DMS increases sampling diversity and facilitates the search for improved subnet architectures.

**Table 8.** The hypervolume comparison of max sampling and double mutation sampling on NSL-KDD, UNSW-NB15, and CIC-IDS2017 datasets

	NSL-KDD		UNSW-NB15		CIC-IDS2017	
	Gen	Dis	Gen	Dis	Gen	Dis
Max sampling	0.002480	0.053376	0.042185	0.000619	3.65E−37	0.106087
Double mutation sampling	0.010577	0.120073	0.042758	0.001284	2.35E−36	0.106098

**Fig. 8.**
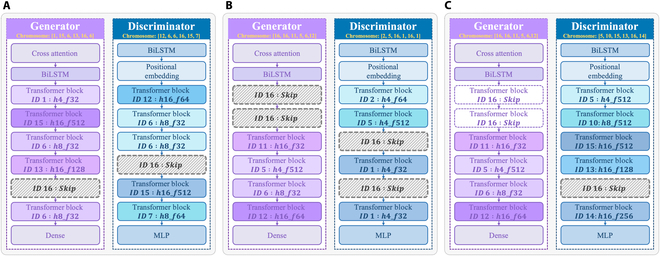
The final GAN architecture of (A) NSL-KDD, (B) UNSW-NB15, and (C) CIC-IDS2017 datasets. The DMM NAS and the fuzzy decision-making method automatically skip specific TBs to achieve a balance between model performance and speed.

Figures 5 to 7 show the optimal generator and discriminator obtained via fuzzy decision-making. The specific structures of these neural network architectures are illustrated in Fig. 8. As shown in Fig. 8 A to C, the final GAN architecture searched from the nondominated solutions of DMM NAS via the fuzzy decision-making method skips specific TBs, which helps reduce redundant computations and achieve the optimal balance between model performance and speed.

### Ablation studies

This section presents the ablation studies of the modules proposed in this paper. To ensure the rigor of the experiments, the parameter settings of the layer number, layer architecture, etc., in the models are kept consistent with those of the previous experiment across all tests. The number of training iterations, optimizer selection, and data preprocessing steps remains the same.

**Fig. 9.**
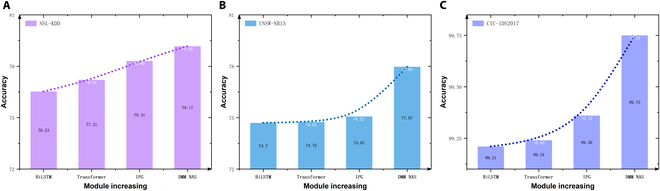
Ablation studies of the multi-class detection accuracy on the (A) NSL-KDD, (B) UNSW-NB15, and (C) CIC-IDS2017 datasets. (Note that a higher value of accuracy is better.) The detection accuracy increases with the increasing of modules.

The ablation study results of the multi-class detection accuracy are shown in Fig. 9. As the number of modules increases, the detection accuracy tends to increase on all the datasets. Comparing the baseline BiLSTM model with the model that includes the Transformer module, the detection accuracy increased by 0.68%, 0.05%, and 0.03% on their respective datasets, revealing that the integration of the attention mechanism in the Transformer enhances the spatiotemporal feature extraction of the Metaverse network traffic. The IPG module improves the detection accuracy on NSL-KDD, UNSW-NB15, and CIC-IDS2017 by 0.90%, 0.32%, and 0.12%, respectively, indicating that the setup of the IPG-based generator helps address the data imbalance issues to improve detection accuracy. Finally, we test the DMM NAS module, and the detection accuracy increases by 0.86%, 2.90%, and 0.39% and up to 79.17%, 77.97%, and 99.75%, respectively. These results validate that our approach effectively enhances the detection accuracy.

**Fig. 10.**
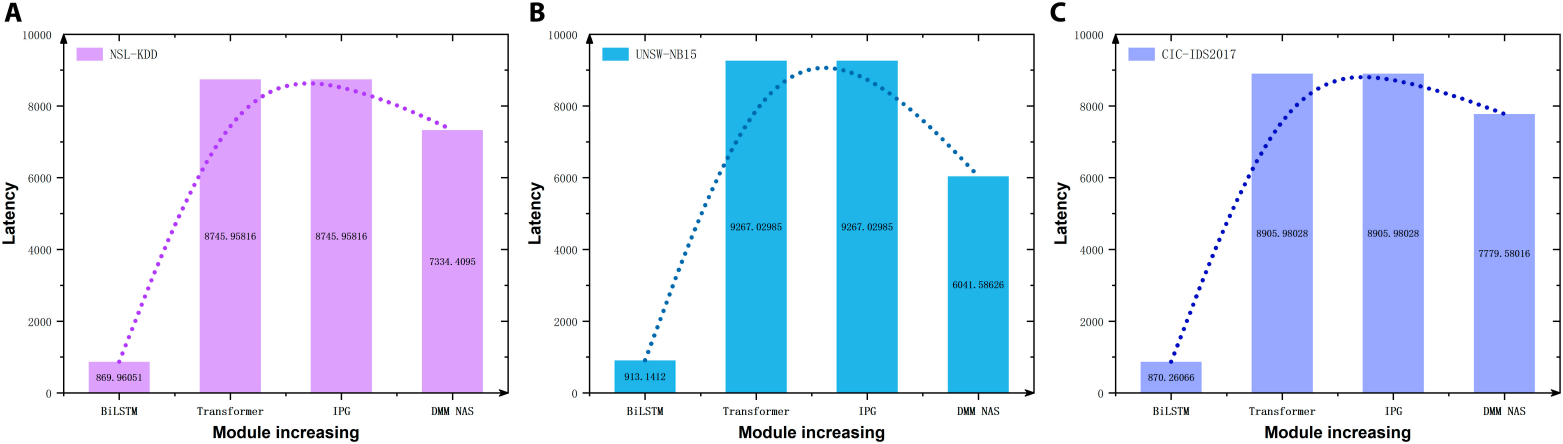
Ablation studies of the calculation latency on the (A) NSL-KDD, (B) UNSW-NB15, and (C) CIC-IDS2017 datasets. (Note that a lower value of accuracy is better.) The detection accuracy increases with the increasing of modules.

The ablation study results of the calculation latency are shown in Fig. 10. The curves for calculation latency differ from those for multi-class detection accuracy as the number of modules increases. Due to the significant consumption of computational resources by the attention mechanism, the introduction of the Transformer module improves model accuracy but also increases computational latency. In contrast, the IPG module improves the distribution of the generator’s sample categories, balancing the minority class data in the training set, which enhances the recognition accuracy. Since this module adjusts only the distribution of the input noise and does not alter the model architecture, its computational latency is comparable to that of the Transformer module. The introduction of the DMM NAS module combines SAES, multiobjective optimization, and NAS, optimizing for both recognition accuracy and computational latency. This enables the search for a nondominated solution set that achieves a good balance between performance and speed. By employing a fuzzy decision method based on the triangular membership function and the weighted average method, the membership matrix is obtained, which is then multiplied by the weighted average matrix to obtain the defuzzification results. Thus, the DMM NAS module can automatically compute and select the optimal solution. The final model obtained through the search optimizes the computational latency introduced by the IPG module and enhances the detection performance.

## Discussion

The deployment of the GAN-based Metaverse NID in this paper benefits from the computational latency provided by the Metaverse device and the AME-GAN on the server side. After determining the basic architecture of the Metaverse NID neural network, the network layers and their candidate blocks will be deployed on the Metaverse device to obtain the real computational latency. To achieve the optimal balance between speed and accuracy of the Metaverse NID, the obtained real computational latency will be applied to the AME-GAN on the server side. The AME-GAN takes the actual computational capabilities of Metaverse devices into account. The IPG strategy of the IPHA LSTM-GAN can generate specific class samples, addressing the randomization flaw in GAN sample generation and the data imbalance issue. The SAES enables the supernet to mutate and generate multiple mutated supernet offspring and adaptively chooses the mutation direction, providing a more robust and stable training. The DMM NAS demonstrates its superiority in enhancing subnet population diversity and optimizing the search for the optimal model. The final optimal model will then be integrated into Metaverse devices for real-time Metaverse traffic monitoring.

Unfortunately, some research limitations remain, requiring further refinement and enhancement. Although the AME-GAN successfully performs the search for the generator and discriminator architectures within a multiobjective GAN framework, it restricts the search to the feature extraction network only, instead of all network layers, to reduce the NAS search space. While the DMS improves the diversity of the sampled subnet population, it also incurs higher computational costs and time consumption. Future work will focus on improving the sampled subnet diversity while reducing its number through methods such as decision variable grouping, achieving similar or improved Metaverse NID performance with minimal increases in search time consumption. Moreover, techniques such as model compression, quantization, and edge computing could be utilized to optimize the deployment of the Metaverse NID model, enabling its efficient operation even on devices with limited hardware capabilities.

## Data Availability

The source code and dataset used in this study can be accessed at https://github.com/TikyXu/AME-GAN.
